# Feline diabetes mellitus – the Swedish situation

**DOI:** 10.1186/1751-0147-57-S1-O13

**Published:** 2015-09-25

**Authors:** Malin Öhlund, Tove Fall, Bodil Ström Holst, Helene Hansson-Hamlin, Brenda Bonnett, Agneta Egenvall

**Affiliations:** 1 Department of Clinical Sciences, Swedish University of Agricultural Sciences, Uppsala, Sweden; 2 Department of Medical Sciences, Uppsala University, Uppsala, Sweden; 3 B. Bonnett Consulting, Georgian Bluffs, Ontario, Canada

## Introduction

Diabetes mellitus (DM) is a common endocrinopathy in cats. Feline DM is similar to human type 2 diabetes (T2D), with obesity as a major risk factor in both cats and humans. Genetics play an important role in the development of obesity and T2D in humans.

## Objectives

The aim was to describe the incidence of feline diabetes in Sweden, and the association with different epidemiologic risk factors.

## Methods

We used data from a cohort of 504,688 cats in a Swedish insurance company from 2009 to 2013, contributing with 1,229,699 cat-years at risk (CYAR). Overall incidence rates (IR) and IRs stratified on age, breed and gender were estimated.

## Results

The IR of diabetes was twelve cases per 10,000 CYAR. Male cats had twice as high IR as females. A significant association with breed was seen, with the Burmese, Russian Blue, Norwegian Forest Cat, and Abyssinian breeds at increased risk. Also domestic cats were at higher risk compared to purebred cats. IRs of DM was highest in cats 12 to 15 years old.

## Conclusions

Our results identified several cat breeds associated with an increased risk of DM. Also male gender and middle age were risk factors for DM. A link has been shown between obesity and DM in cats. A higher incidence of obesity has been reported in domestic cats as compared to purebreds, in male cats, and in middle-aged cats; these groups are similar to those in which we identified a higher occurrence of DM.

